# Impact of obesity on pathological complete remission in early stage breast cancer patients after neoadjuvant chemotherapy: a retrospective study from a German University breast center

**DOI:** 10.1007/s00404-024-07786-7

**Published:** 2024-10-28

**Authors:** Johannes Felix Englisch, Alexander Englisch, Dominik Dannehl, Kenneth Eissler, Christian Martin Tegeler, Sabine Matovina, Léa Louise Volmer, Diethelm Wallwiener, Sara Y. Brucker, Andreas Hartkopf, Tobias Engler

**Affiliations:** Department of Women’s Health, Tübingen University, 72076 Tübingen, Germany

**Keywords:** Early-stage breast cancer, Neoadjuvant chemotherapy, Pathological complete response (pCR), Obesity, Body mass index (BMI)

## Abstract

**Purpose:**

Breast cancer is a primary cause of cancer-related death among women worldwide. Neoadjuvant chemotherapy (NACT) is a cornerstone treatment for locally advanced, non-metastatic breast cancer. Achieving pathological complete response (pCR) is often used as a surrogate marker for long-term outcomes. This study examines the impact of obesity, defined by a body mass index (BMI) over 30 kg/m^2^, on achieving pCR in patients with early stage breast cancer (eBC) undergoing NACT.

**Methods:**

A retrospective analysis was conducted on patients with eBC treated with NACT at the University of Tübingen. The primary objective was to assess the impact of obesity on achieving pCR. Logistic regression analysis was used to determine the association between pre-treatment BMI and pCR, adjusting for covariates such as age, tumor stage, grading, and chemotherapy intensity.

**Results:**

The study included 325 patients, with 24% classified as obese. While the univariate logistic regression analysis showed no significant impact of obesity on the odds ratio of achieving pCR, the multivariate analysis, accounting for covariates, demonstrated that obese patients had a significantly higher likelihood of achieving pCR.

**Conclusion:**

In this retrospective study, obesity significantly affected the likelihood of achieving pCR in patients with eBC cancer undergoing NACT after adjusting for covariates. While obesity is a known risk factor for breast cancer development and progression, its impact on the efficacy of NACT in terms of achieving pCR was positive in our study. These findings contribute to the ongoing debate in the literature, though the retrospective design and potential uncontrolled factors should be considered.

**Supplementary Information:**

The online version contains supplementary material available at 10.1007/s00404-024-07786-7.

## What does this study add to the clinical work

**Table:** 

Although obesity is a known risk factor for breast cancer, our study found that it positively impacts the efficacy of NACT in achieving pCR. This finding is encouraging for treating the increasing number of obese patients with early breast cancer.	

## Introduction

Breast cancer is a leading cause of cancer-related morbidity and mortality among women worldwide. Neoadjuvant chemotherapy (NACT) has become a standard treatment for locally advanced, non-metastatic early breast cancer (eBC), playing a crucial role in tailoring further adjuvant therapy for high-risk patients. Achieving pathological complete response (pCR) after NACT is a key indicator of favorable long-term outcomes, particularly in aggressive subtypes like triple-negative or human epidermal growth factor receptor 2 (HER2)-positive breast cancer [1–4]. Consequently, research has focused on identifying clinicopathological features that predict pCR in patients receiving NACT.

In addition to molecular subtypes and tumor stage, constitutional factors such as obesity are being explored as potential predictors for pCR [5, 6]. As obesity increasingly threatens public health, it is important to consider its impact on the risk of developing breast cancer and its effect on treatment [7]. Studies have shown that obesity, defined by a body mass index (BMI) over 30 kg/m^2^, increases the risk of developing breast cancer, especially hormone receptor-positive types [8–10]. In early stage breast cancer, obesity is associated with advanced tumor stages and reduced overall and breast cancer-specific survival [11, 12]. There are contradictory studies in the current literature regarding the relationship between BMI and achieving pCR after NACT, with some research suggesting that higher BMI may correlate with lower pCR rates, while other studies found no significant association [5, 6]. This indicates the complex role of obesity in breast cancer prognosis and treatment response, highlighting the need for further research.

To better understand the impact of obesity on achieving pCR and gain valuable insights for optimizing therapeutic strategies for obese patients, this retrospective study conducted at a large German university breast center aims to elucidate the impact of obesity on achieving pCR in patients with eBC. By examining tumor characteristics and chemotherapy protocols across a uniformly treated eBC cohort and employing logistic regression analysis to account for relevant covariates, the study seeks to provide a comprehensive understanding of how obesity influences the likelihood of reaching pCR. The use of real-world data from a single cancer center, where all patients received care under consistent conditions, enhances the practical relevance of the findings.

## Methods

### Study design and treatment protocols

This retrospective analysis included patients with early invasive breast cancer treated with neoadjuvant chemotherapy at the University of Tübingen, Germany, from January 2014 to December 2021. Patient medical records, pharmacy dispensing records, and histopathology reports were reviewed to extract information on treatment regimens administered, dosages, and any treatment delays. Patients were included based on the planned chemotherapy protocol prior to the initiation of treatment. Eligible patients received at least one cycle of one of the chemotherapy regimens described in Supplementary Table 1. Patients with HER2-positive disease may have received anti-HER2 therapy, including Trastuzumab alone or in combination with Pertuzumab. Patients with triple-negative breast cancer (TNBC) may have received PD-1 checkpoint inhibitor therapy with Pembrolizumab. Patients who received other chemotherapies in addition to the ones mentioned in Supplementary Table 1 were excluded from the study. Male patients and individuals with metastatic disease were excluded as well. In cases of bilateral breast cancer, the tumor with greater clinical significance was considered for analysis.

### Variables

Pathologic complete response (pCR) was the primary endpoint of this study to measure neoadjuvant treatment efficacy and was defined as the elimination of detectable invasive cancer in the breast and lymph nodes (ypT0/ypTis and ypN0) at the time of surgery [3]. The primary variable of interest was the pre-treatment BMI, with patients categorized as obese (BMI ≥ 30 kg/m^2^) or non-obese (< 30 kg/m^2^) according to the criteria of the World Health Organization [7]. Tumors were classified as estrogen receptor (ER) positive if more than 10% of cells were ER positive or, if ER percentage was not measured, by an immunoreactive score (IRS) of at least 1. HER2-positive tumors were identified with an immunohistochemistry (IHC) score of 3 + or a score of 2 + and amplified fluorescence in situ hybridization (FISH) or chromogenic in situ hybridization (CISH) result. Relative dose intensity (RDI) was used as the primary measure of chemotherapy treatment intensity to account for delays, dose reductions, and treatment discontinuations. Calculation of RDI was based on the actual body surface area as calculated by the DuBois formula [13]. RDI was defined as delivered dose intensity (DDI) divided by standard dose intensity (SDI). DDI was calculated as (delivered dose, in mg/m^2^) / (actual time to complete therapy plus standard time required for remaining missed cycles, in days). SDI was calculated as (planned dose, in mg/m^2^) / (standard time to complete chemotherapy, in days) [14, 15]. For chemotherapy regimens containing multiple agents, the RDI was calculated individually for each drug before an average was calculated for the entire regimen. For patients receiving multiple chemotherapy regimens, the mean RDI for all regimens was calculated. If a second regimen was planned but not administered, we considered the RDI of the second regimen to be zero. Antibody components against HER2 and immunotherapies were not included in the calculation of RDI.

### Statistical analysis and data visualization

Jupyter Notebooks (Jupyter core 5.3.1, Python 8.14.0) and Python 3.11.7 were used for data analysis and visualization. Statistical methods such as *t* test for normally distributed data, Mann–Whitney U test for non-normal distributions, and Fisher’s exact test for ordinal data were employed as detailed in tables and figure legends. The normality of data distribution was assessed using the Shapiro–Wilk test. For the logistic regression analysis and calculation of odds ratios, key variables such as the AJCC anatomical stage and tumor grading were numerically coded. Binary variables were established for obesity status, treatment response (pCR), menopausal status, and ER and HER2 status. The logistic regression model was fitted using the statsmodels library (Version 0.14.0), employing a Logit model with maximum likelihood estimation (MLE) to fit the data. A constant was added to the model to ensure that it accurately reflects the data structure. Odds ratios were calculated from the regression coefficients to quantify the association between these variables and the likelihood of achieving pCR. This analysis was supported by the following Python packages: pandas (Version 2.1.4), matplotlib (Version 3.8.0) and seaborn (Version 0.12.2), NumPy (Version 1.26.4), statsmodels.api (Version 0.14.0) and scipy.stats (Version 1.11.4).

## Results

### Patient characteristics

Three hundred twenty-five patients were included in the study, with two hundred forty-seven (76.0%) classified as non-obese and seventy-eight (24.0%) as obese, based on a BMI threshold of ≥ 30 kg/m^2^. Baseline characteristics of non-obese and obese patients were similar, as shown in Table 1. The mean age ± SD was 52.23 ± 11.11 years for non-obese and 54.21 ± 11.68 years for obese patients. The proportion of pre- and post-menopausal patients was comparable between the groups. The non-obese group had a median BMI of 24.05 kg/m^2^ (range: 16.26–29.74 kg/m^2^) and the obese group had a median BMI of 35.10 kg/m^2^ (range: 30.04–51.99 kg/m^2^). There were no significant differences in histological tumor subtype, grading, nodal status, ER status, and HER2 status among the groups. The obese group had a higher incidence of T2–4 stage tumors (p = 0.035) and a higher simplified AJCC-Stage (*p* = 0.029) compared to the non-obese group.

**Table 1:** Patient characteristics

	Non-obese (*n* = 247)	Obese (*n* = 78)	*p* value	Test
Age in years	52.23 ± 11.11	54.21 ± 11.68		t test
BMI in kg/m^2^	24.05 (16.26–29.74)	35.10 (30.04–51.99)	< 0.001	Mann–Whitney U
Menopausal status				
Postmenopausal	117 (47.4%)	41 (52.6%)	0.438	Fisher’s exact
Premenopausal	130 (52.6%)	37 (47.4%)		
Histology				
NST	227 (91.9%)	68 (87.2%)		Fisher’s exact
ILC	9 (3.6%)	4 (5.1%)		
Other	11 (4.5%)	6 (7.7%)		
Grading				
G1	1 (0.4%)	2 (2.6%)	0.263	Fisher’s exact
G2	92 (37.2%)	28 (35.9%)		
G3	154 (62.4%)	48 (61.5%)		
Clinical T stage (initial)				
T1	57 (23.1%)	9 (11.5%)	0.035	Fisher’s exact
T2-4	190 (76.9%)	69 (88.5%)		
Clinical N stage (initial)				
N0	163 (66.0%)	42 (53.8%)	0.060	Fisher’s exact
N1-3	84 (34.0%)	36 (46.2%)		
Simplified AJCC stage (initial)				
Stage I	38 (15.4%)	6 (7.7%)	0.029	Fisher’s exact
Stage II	159 (64.4%)	46 (59.0%)		
Stage III	50 (20.2%)	26 (33.3%)		
ER status				
Negative	83 (33.6%)	26 (33.3%)	> 0.999	Fisher’s exact
Positive	164 (66.4%)	52 (66.7%)		
HER2 status				
Negative	114 (46.2%)	35 (44.9%)	0.897	Fisher’s exact
Positive	133 (53.8%)	43 (55.1%)		

### Neoadjuvant therapy received

To further compare obese and non-obese patients, we analyzed therapy received, including the use of different chemotherapeutic drugs, anti-HER2 and checkpoint-inhibitor therapies. We found no significant difference between the two groups, with a p value of 0.630, as shown in Table 2. We also compared the planned neoadjuvant regimens as indicated in Supplementary Table 1, finding no significant difference with a p value of 0.598.

**Table 2:** Neoadjuvant therapy received

	Non-obese (*n* = 247)	Obese (*n* = 78)	p value (Fisher’s exact test)
Therapy received			
Anthracycline	205 (83.0%)	65 (83.3%)	0.630
Cyclophosphamide	205 (83.0%)	65 (83.3%)	
Platinum	42 (17.0%)	10 (12.8%)	
Taxan	239 (96.8%)	77 (98.7%)	
Anti-HER2	130 (52.6%)	42 (53.8%)	
Checkpoint-Inhibitor	4 (1.6%)	4 (5.1%)	

### Logistic regression analysis

**Fig. 1:**
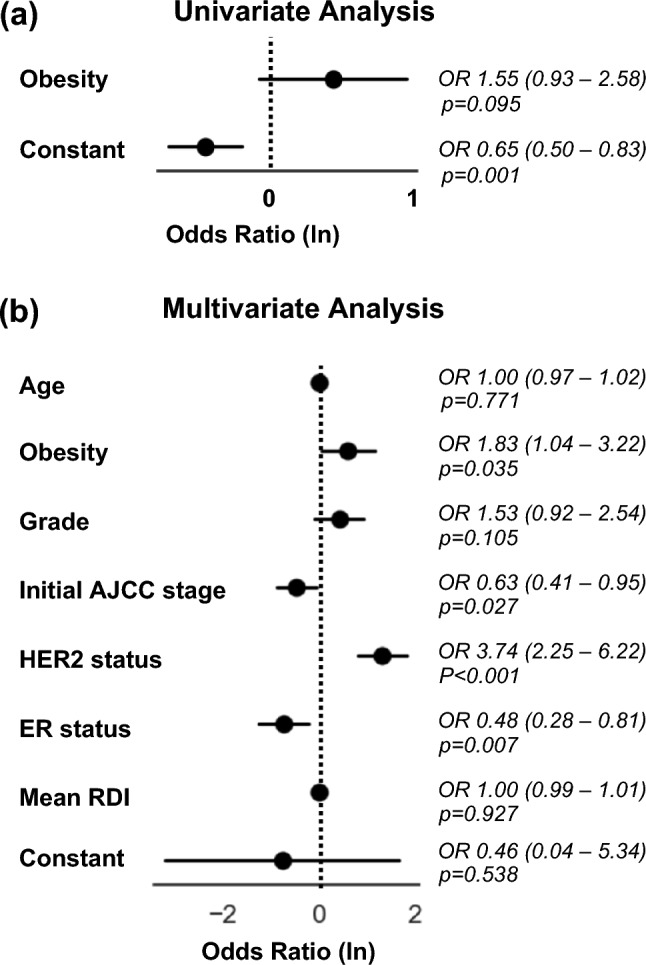
Logistic regression analysis of pathologic complete response after neoadjuvant chemotherapy

To investigate the impact of pre-treatment BMI on the probability of achieving complete treatment response (pCR), a univariate analysis was performed. This analysis revealed an odds ratio of 1.55 for obese (BMI ≥ 30 kg/m^2^) compared to non-obese patients to achieve a pCR, with a confidence interval ranging from 0.93 to 2.58 and a *p* value of 0.095. The model achieved a log-likelihood of −219.54 with a pseudo R-squared of 0.006291, indicating a limited explanatory power of BMI on treatment outcomes (Fig. 1).

Given the limited insight provided by the univariate analysis, a multivariate logistic regression analysis was conducted to evaluate the influence of BMI and other clinical and pathological variables on pCR. This analysis incorporated age, initial AJCC anatomical stage, tumor grading, mean relative dose intensity (mean RDI), ER status, and HER2 status as covariates. These variables were selected to accurately reflect tumor biology, stage, and chemotherapy intensity, following the Chemotherapy Response Calculator for Neoadjuvant Therapy of Breast Cancer at MD Anderson Cancer Center [16]. The multivariate analysis showed that obese patients had an odds ratio of 1.83 for achieving pCR, with a confidence interval from 1.04 to 3.22, and a *p* value of 0.035, indicating a significant positive impact. In addition, the analysis identified other significant predictors of pCR: HER2-positive status, which had an odds ratio of 3.74 (confidence interval: 2.25 to 6.22, *p* value: < 0.001); ER positive status, with an odds ratio of 0.48 (confidence interval: 0.28 to 0.81, *p* value: 0.007); and initial AJCC stage, with an odds ratio of 0.63 (confidence interval: 0.41 to 0.95, *p* value: 0.027). The model’s improved log-likelihood of − 196.32 and a pseudo R-squared of 0.1114 suggest enhanced predictive capacity for treatment outcomes.

## Discussion

Our study evaluated the impact of obesity on achieving pCR after NACT in patients with early breast cancer. Initially, we demonstrated uniformity in baseline characteristics across patient groups, including histological tumor subtype, grading, nodal status, ER status, HER2 status, and the chemotherapeutic and antibody medications administered. Interestingly, despite obese patients more likely presenting with advanced tumor stages (T2-4 and higher AJCC stages), our multivariate regression model—adjusting for age, initial AJCC stage, tumor grading, and mean relative dose intensity—identified obesity as a significant positive predictor of pCR.

Our findings contribute to the complex discourse on the impact of BMI on treatment responses in breast cancer. While obesity is associated with improved survival in the metastatic setting, meta-analyses of published studies report poorer survival outcomes for obese patients with eBC, a phenomenon often referred to as the “obesity paradox” [17–19]. In our study, we selected the achievement of pCR as the endpoint, a well-established prognostic marker for patients with early breast cancer [20]. Recently, pCR has become increasingly critical in determining adjuvant therapy strategies. Patients who do not achieve pCR now have access to new and innovative medications that can significantly enhance prognosis and survival [21].

Motivated by conflicting reports in existing literature regarding whether obese patients are more or less likely to achieve pCR, our study aimed to explore if obese patients, potentially exhibiting lower rates of pCR, might gain quicker access to innovative treatment options. Contrary to this hypothesis, and contrary to findings from Wang et al. [6], our data revealed that obese patients demonstrated a higher rate of pCR. This suggests that obese patients do not experience a disadvantage in neoadjuvant chemotherapy effectiveness and indicates that the premise of obese patients having quicker access to newer treatments due to poor initial response does not hold.

Several factors might explain the higher rate of pCR observed in obese patients. One theory is that obesity might be associated with increased proliferation of tumor cells in patients with hormone receptor-positive/HER2-negative tumors, enhancing their chemotherapy susceptibility due to hormonal proliferation stimuli from increased adipose tissue. The relatively small size of our patient cohort and the absence of retrospective data, e.g., on estrogen serum levels limited detailed subgroup analyses and further exploration of these interactions. Other potential explanatory factors include variations in pharmacokinetics and alterations in drug metabolism due to differences in body composition among obese patients. As a confounder for pCR, we analyzed the used neoadjuvant treatment regimens, as these could possibly be altered due to a higher spectrum of comorbidities in obese patients. Evaluating the data, we found that the drugs and antibodies administered were comparable between obese and non-obese patients, allowing us to compare differences in real-world treatment admissions.

## Conclusion

In summary, our study demonstrated significantly better pCR rates after NACT in obese patients compared to non-obese patients, challenging existing narratives. The strength of our study lies in the uniform distribution of clinical and pathological characteristics, enabling reliable comparisons and minimizing confounders. The consistent application of chemotherapy regimens across patient groups further supports the validity of our findings. These results encourage us in dealing with the ever-growing population of obese patients with early breast cancer. However, due to the limitations of the retrospective study design and the small cohort size, further studies are necessary to better evaluate the causes of differential therapy responses and to enable detailed subgroup analyses.

## Supplementary Information

Below is the link to the electronic supplementary material.

Supplementary file1 (DOCX 41 KB)

## Data Availability

The datasets generated and analyzed during the current study are not publicly available due to restrictions related to patient confidentiality and privacy but are available from the corresponding author on reasonable request, subject to necessary ethical approvals. However, the Python code used for the analysis is available upon request to ensure transparency and reproducibility of the study's findings. Interested researchers may contact the corresponding author for access to the code.

## Abbreviations

*NACT*: Neoadjuvant chemotherapy
*AJCC*: American joint committee on cancer
*pCR*: Pathological complete response
*eBC*: Early stage breast cancer
*BMI*: Body mass index
*HER2*: Human epidermal growth factor receptor 2
*TNBC*: Triple negative breast cancer
*ER*: Estrogen receptor
*IRS*: Immunoreactive score
*IHC*: Immunohistochemistry
*FISH*: Fluorescence in situ hybridization
*CISH*: Chromogenic in situ hybridization
*RDI*: Relative dose intensity
*DDI*: Delivered dose intensity
*SDI*: Standard dose intensity
*MLE*: Maximum likelihood estimation
