# Supplementary material for: Impact of obesity on pathological complete remission in early stage breast cancer patients after neoadjuvant chemotherapy: a retrospective study from a German University breast center
Source: Arch Gynecol Obstet. 2024 Oct 28;311(2):437–42. doi: 10.1007/s00404-024-07786-7 (PMC11890308; doi:10.1007/s00404-024-07786-7)
Supplement: Supplementary file 1 — Supplementary file1 (DOCX 41 KB) [file 404_2024_7786_MOESM1_ESM.docx]

**Impact of Obesity on Pathological Complete Remission in Early-Stage Breast Cancer Patients after Neoadjuvant Chemotherapy: A Retrospective Study from a German University Breast Center**

**Journal: Archives of Gynecology and Obstetrics**

Authors: Johannes Felix Englisch, Alexander Englisch, Dominik Dannehl, Kenneth Eissler, Christian Martin Tegeler, Sabine Matovina, Léa Louise Volmer, Diethelm Wallwiener, Sara Y. Brucker, Andreas Hartkopf, Tobias Engler

Affiliations: Department of Women's Health, Tübingen University, 72076 Tübingen, Germany

## Corresponding author contact information: Johannes.englisch@med.uni-tuebingen.de

# Supplement

**Supplementary Table 1: Neoadjuvant Therapy Sequence**

|  | **non-obese (n=247)** | **obese (n=78)** | **p-Value (Fisher’s exact)** |
| --- | --- | --- | --- |
|  |  |  |  |
| **Regime received** |  |  | **0.598** |
| Anthracycline / Cyclophosphamid↔ Anti-HER2↔ Taxan | 99 (40.1%) | 32 (41.0%) |  |
| Anthracycline / Cyclophosphamid↔ Taxan | 74 (30.0%) | 22 (28.2%) |  |
| Anthracycline / Cyclophosphamid↔ Carboplatin / Taxan | 21 (8.5%) | 6 (7.7%) |  |
| Anti-HER2↔ Taxan | 19 (7.7%) | 8 (10.2%) |  |
| Anti-HER2↔ Carboplatin / Taxan | 12 (4.9%) | 2 (2.6%) |  |
| Carboplatin / Taxan | 9 (3.6%) | 2 (2.6%) |  |
| Anthracycline / Cyclophosphamid | 8 (3.2%) | 1 (1.3%) |  |
| Anthracycline / Cyclophosphamid↔ Checkpoint-Inhibitor | 3 (1.2%) | 4 (5.1%) |  |
| Taxan | 1 (0.4%) | 1 (1.3%) |  |
| Checkpoint-Inhibitor↔ Taxan | 1 (0.4%) | 0 (0%) |  |
